# Gender-Associated Outcomes Following Percutaneous Coronary Intervention With a Third-Generation, Ultrathin-Strut Drug-Eluting Stent: A Real-World, Single-Center Experience

**DOI:** 10.3389/fcvm.2021.796604

**Published:** 2022-02-11

**Authors:** Sebastiano Gili, Stefano Galli, Giovanni Teruzzi, Giulia Santagostino Baldi, Paolo Ravagnani, Franco Fabbiocchi, Antonio Bartorelli, Piero Montorsi, Daniela Trabattoni

**Affiliations:** ^1^Centro Cardiologico Monzino, Istituto di Ricovero e Cura a Carattere Scientifico (IRCCS), Milan, Italy; ^2^Department of Biomedical and Clinical Sciences, “Luigi Sacco”, University of Milan, Milan, Italy; ^3^Department of Clinical Sciences and Community Health, University of Milan, Milan, Italy

**Keywords:** ultrathin-strut, bioresorbable-polymer, drug-eluting stents, outcomes, PCI in women

## Abstract

**Introduction:**

In recent years, the new third-generation ultrathin bioresorbable-polymer sirolimus-eluting stent (BP-SES), characterized by some of the thinnest struts among commercially available devices (60–80 μm) and an amorphous silicon carbide coating, has been introduced for the treatment of coronary artery disease (CAD). The present study aimed to assess different clinical outcomes and safety of this drug-eluting stent in male and female patients in a real-world setting.

**Methods:**

The present study is a retrospective analysis including all patients treated with BP-SES between January 2017 and December 2019 at a single high-volume center. Follow-up data, including stress test results and clinical setting, were collected during outpatient visits or by telephone contact. Patients symptomatic for angina or with a positive stress test were addressed to CT scan/coronary angiogram. The main study outcome was target lesion failure (TLF), defined as a composite of cardiovascular death, target vessel myocardial infarction, or target lesion revascularization.

**Results:**

Overall, 66 (15.9%) female and 349 (84.1%) male patients were included; women were older (median age 70 vs. 66, *P* = 0.003) and with a lower body mass index (BMI) (25.0 vs. 26.1, *P* = 0.010) compared to men, with no other relevant differences in baseline characteristics. Indication for percutaneous coronary intervention (PCI) was acute coronary syndrome in 86 (20.7%) of the cases, with no significant differences between male and female patients. A total of 558 lesions were treated with BP-SES stents, 90 in women and 468 in men (1.36 vs. 1.34 lesions per patient, *P* = 0.83); cumulative stent length (33.6 vs. 38.4 mm, *P* = 0.078), and mean stent diameter (2.92 vs. 3.0 mm, *P* = 0.39) did not differ in women compared to men. Technical and clinical successes were achieved in all patients. Stent thrombosis (ST) occurred in 2 (0.5%) patients, both men. TLF occurred in 10 (2.9%) men and 2 (3.0%) women after a median follow-up of 402 days, without significant differences at log-rank analysis (2.34 events per 100 patient-years in men, 2.53 in women; *P* = 0.80).

**Conclusion:**

Ultrathin struts BP-SES showed to be a safe and effective option for the treatment of CAD in both women and men, with a very low ST rate and favorable long-term outcomes.

## Introduction

Women have been historically underrepresented in randomized clinical trials, particularly in the cardiovascular field. Newly developed medical devices and drugs are too often tested in unbalanced populations in terms of gender in controlled studies, which are hardly representative of the real-world populations in which such devices are used (1). This limitation applies as well to stent technology, a field in which the evolution from bare-metal stents (BMS) to first and second-generation drug-eluting stents (DES) has led to significant prognostic improvements in women in terms of adverse cardiovascular events at follow-up (2). During the course of the years, several studies have suggested that women might experience worse outcomes compared to men following percutaneous coronary intervention (PCI), in particular with earlier second-generation drug-eluting stents (3, 4), even if potentially not because of stent-related events (5). These findings have been however inconsistent and conflicting with other studies failing to recognize such differences (6).

Research and development of coronary DES have led to marked improvements in several stent technology features, from the type of eluted-drugs to polymer and struts design as well. Stent struts thickness has been progressively reduced, with favorable effects in terms of timing for endothelialization, local inflammation, vessel injury, thrombogenicity, and neointimal proliferation (7, 8). Introduction of bioresorbable polymers (BP), which progressively disappear after implantation, has led to the development of DES transforming over time into biologically less active BMS, with a potential reduction of long-term thrombogenicity (9). These technological improvements have guided the creation of a recently distributed ultrathin BP sirolimus-eluting stent (BP-SES), with some of the thinnest struts among commercially available devices (60–80 μm based on stent diameter) and an amorphous silicon carbide coating, which has shown promising results in early randomized clinical trials (RCT) (10).

With the increasing use of ultrathin BP-SES in clinical practice, it is of utmost importance to test the safety and effectiveness of such devices different among gender in a real-world clinical setting, especially because patients treated in routine clinical practice may often present different features and offer different challenges compared to those included in RCT. The present study aims therefore to evaluate the short and mid-term outcomes in women and men treated with the implantation of ultrathin BP-SES during routine clinical practice.

## Methods

The present study is a single-center, retrospective analysis including all patients treated with the implantation of at least one ultra-thin BP-SES between January 2017 and December 2019. All coronary interventions were performed at Centro Cardiologico Monzino, IRCCS, Milan, Italy, a third-level high-volume cardiologic referral center. Choice of the type of implanted stents was left at the operator's discretion. No formal exclusion criteria were defined.

For all patients, baseline clinical features, past medical history, indications for PCI, and main laboratory values were collected. Coronary anatomical characteristics, lesion features, and procedural data were registered as well. Chronic coronary syndrome, non-stent thrombosis (ST) elevation acute coronary syndrome [NSTE-ACS, comprising patients with non-ST elevation myocardial infarction (NSTEMI) and unstable angina (UA)], and ST-elevation myocardial infarction (STEMI) were defined according to the current European Society of Cardiology guidelines (11–13). Technical success was defined as a successful lesion dilation and stent deployment in the target coronary segment, with a final Thrombolysis in Myocardial Infarction (TIMI) flow of three and residual stenosis of <30%. Clinical success was the achievement of technical success without procedure-related adverse clinical events. Periprocedural myocardial infarction (PMI) was defined according to the Fourth Universal Definition of Myocardial Infarction (14). Medical treatment and periprocedural management were carried out according to current guidelines and good clinical practice principles. A small vessel PCI subgroup included patients in whom the diameter of the largest implanted BP-SES was 2.5 mm.

In hospital and post-discharge, medical treatment was prescribed according to current guidelines, in particular, all patients were on at least one antiplatelet drug before stent implantation. Clopidogrel with a loading dose of 600 mg and a maintenance 75 mg daily dose for 6 months was administered on top of aspirin in patients with chronic coronary syndromes, whereas patients with the acute coronary syndrome (ACS) were given a loading dose of Prasugrel or Ticagrelor followed by a maintenance dose of the respective drug for 12 months.

All patients were prescribed a stress test 8–10 months after PCI and then yearly. Follow-up data were collected during the outpatient visit or, whereas not available, by telephone contact. Patients symptomatic for angina or with a positive stress test were addressed to CT scan/coronary angiogram. The main outcome assessed was target lesion failure (TLF), defined as a composite of cardiovascular death, target vessel myocardial infarction (MI), or target lesion revascularization (TLR, defined as revascularization–either by PCI or coronary artery bypass graft–of stenosis occurring within the stent or within 5 mm proximal or distal to the stent edge). Secondary outcomes evaluated comprised individual components of the composite outcome, all-cause death, and stent thrombosis, defined according to the Academic Research Consortium criteria.

### Statistical Analysis

Continuous variables were reported as median with interquartile ranges (IQR) or mean ± SD and were compared by Kruskal-Wallis non-parametric test; categorical variables are reported as numbers with percentages and were compared by Pearsons's Chi-square test or Fisher's exact test, as appropriate.

Event rates are reported as per 100 patient-years; survival analyses were evaluated by Kaplan Meier curves and were compared by log-rank test.

A two-sided *p*-value of 0.05 was set as statistically significant. All analyses were performed using Jamovi version 1.6.21.0.

## Results

Overall, 416 patients were included, of whom 66 were of female gender; women were older (70 vs. 66 years, *P* = 0.003) and presented lower body mass index (BMI) (25.0 vs. 26.1, *P* = 0.010) compared to men, whereas other baseline clinical features were similar between genders (Table 1). Clinical indication for PCI was chronic coronary syndrome in 329 (79.3%) patients, ST-elevation MI (STEMI) in 51 (12.3%), non-STEMI in 26 (6.3%), and unstable angina in 9 (2.2%) cases, with a similar distribution in men and women.

**Table 1:** Baseline clinical features and in-hospital outcomes.

	**Overall**	**Men**	**Women**	***P*-value**
	**(*N* = 415)**	**(*N* = 349)**	**(*N* = 66)**	
**Age, yrs (range)**	67 (60–74)	66 (59–73)	70 (65–75)	0.003
BMI	26.0 (24.2–28.0)	26.1 (24.4–28.2)	25.0 (23.1–27.2)	0.010
Follow-up length, days (range)	402 (256–618)	400 (264–619)	408 (224–608)	0.50
Hypertension, *n* (%)	290 (69.9)	241 (69.1)	49 (74.2)	0.40
Familal history of CAD, *n* (%)	63 (15.2)	55 (15.8)	8 (12.1)	0.45
Smoke
Active, *n* (%)	40 (9.6)	36 (10.3)	4 (6.1)	0.28
Former, *n* (%)	78 (18.8)	67 (19.2)	11 (16.7)	0.63
Dyslipidemia, *n* (%)	281 (67.7)	239 (68.5)	42 (63.6)	0.44
Diabetes mellitus, *n* (%)	69 (16.6)	63 (18.1)	6 (9.1)	0.073
Non-insulin dependent, *n* (%)	62 (14.9)	57 (16.3)	5 (7.6)	0.067
Insulin dependent, *n* (%)	7 (1.7)	6 (1.7)	1 (1.5)	0.91
Chronic kidney disease, *n* (%)	6 (1.4)	6 (1.7)	0 (0)	0.28
Previous MI, *n* (%)	43 (10.4)	38 (10.9)	5 (7.6)	0.42
Previous PCI, *n* (%)	134 (32.3)	119 (34.1)	15 (22.7)	0.070
Previous CABG, *n* (%)	40 (9.6)	37 (10.6)	3 (4.5)	0.13
Clinical presentation				0.34
STEMI, *n* (%)	51 (12.3)	44 (12.6)	7 (10.6)	
NSTEMI, *n* (%)	26 (6.3)	20 (5.7)	6 (9.1)	
Unstable angina, *n* (%)	9 (2.2)	6 (1.7)	3 (4.5)	
Stable CAD, *n* (%)	329 (79.3)	279 (79.9)	50 (75.8)	
Periprocedural MI, *n* (%)	51 (12.2)	39 (11.2)	12 (18.2)	0.12

*Values are expressed as mean ± standard deviation or number (%), as appropriate*.

*BARC, Bleeding academic research consortium; CABG, coronary artery bypass graft; CAD, coronary artery disease; LAD, left anteriore descending coronary; LCx, left circumflex coronary; MI, myocardial infarction; NSTEMI, non ST-elevation myocardial infarction; PCI, percutaneous coronary intervention; STEMI, ST-elevation myocardial infarction*.

As reported in Table 2, out of a total of 609 lesions, 558 were treated with the implantation of at least one BP-SES DES (1.36 lesions per patient in women, 1.34 in men, *P* = 0.83), whereas 51 lesions were treated with a different kind of DES. Mean stent diameter was similar across genders (2.92 ± 0.42 in women vs. 3.00 ± 0.41 mm in men, *P* = 0.39), whereas mean cumulative stent length was borderline longer in men (33.6 ± 21.4 vs. 38.4 ± 22.9 mm in men, *P* = 0.078). No differences in the rate of small vessel PCI were observed (47.0 vs. 40.7%, *P* = 0.34). Technical and clinical successes were achieved in all patients; no in-hospital deaths were recorded.

**Table 2:** Procedural clinical features and in-hospital outcomes.

	**Overall**	**Men**	**Women**	***P*-value**
	**(*N* = 415)**	**(*N* = 349)**	**(*N* = 66)**	
N° treated lesions	609	510	99	
N° treated lesions per patient	1.47	1.46	1.50	0.99
N° lesions treated w/Orsiro, *n*	558	468	90	
N° lesions treated w/Orsiro per patient	1.34	1.34	1.36	0.83
Treated vessel^*^				0.28
Left Main, *n* (%)	30 (4.9)	27 (5.3)	3 (3.0)	
Left anterior descending, *n* (%)	220 (36.1)	178 (34.9)	42 (42.4)	
Left circumflex, *n* (%)	134 (22.0)	112 (22.0)	22 (22.2)	
Right coronary artery, (%)	152 (25.0)	133 (26.1)	19 (19.2)	
Other vessels, *n* (%)	17 (2.8)	16 (3.1)	1 (1.0)	
Small vessel (diameter ≤ 2. 5 mm), *n* (%)	173 (41.7)	142 (40.7)	31 (47.0)	0.34
N° Orsiro stents per patient				0.13
1 stent, *n* (%)	185 (44.6)	149 (42.7)	36 (54.5)	
2 stents, *n* (%)	115 (27.7)	100 (28.7)	15 (22.7)	
≥ 3 stents, *n* (%)	113 (27.2)	98 (28.1)	15 (22.7)	
Non orsiro stents				0.16
1, *n* (%)	46 (11.1)	35 (10.0)	11 (16.7)	
2, *n* (%)	17 (4.1)	17 (4.9)	0	
≥ 3, *n* (%)	10 (2.4)	7 (2.0)	3 (4.5)	
Cumulative orsiro stent length, mm (mean, ±SD)	37.6 (± 22.7)	38.4 (± 22.9)	33.6 (± 21.4)	0.078
Mean orsiro stent diameter, mm (mean, ±SD)	2.99 (± 0.42)	3.00 (± 0.41)	2.92 (± 0.42)	0.39
Side branch stenting, *n* (%)	21 (5.1)	20 (5.7)	1 (1.5)	0.15

* *percentage expressed in relation to the total number of treated lesions*.

**Figure 1:**
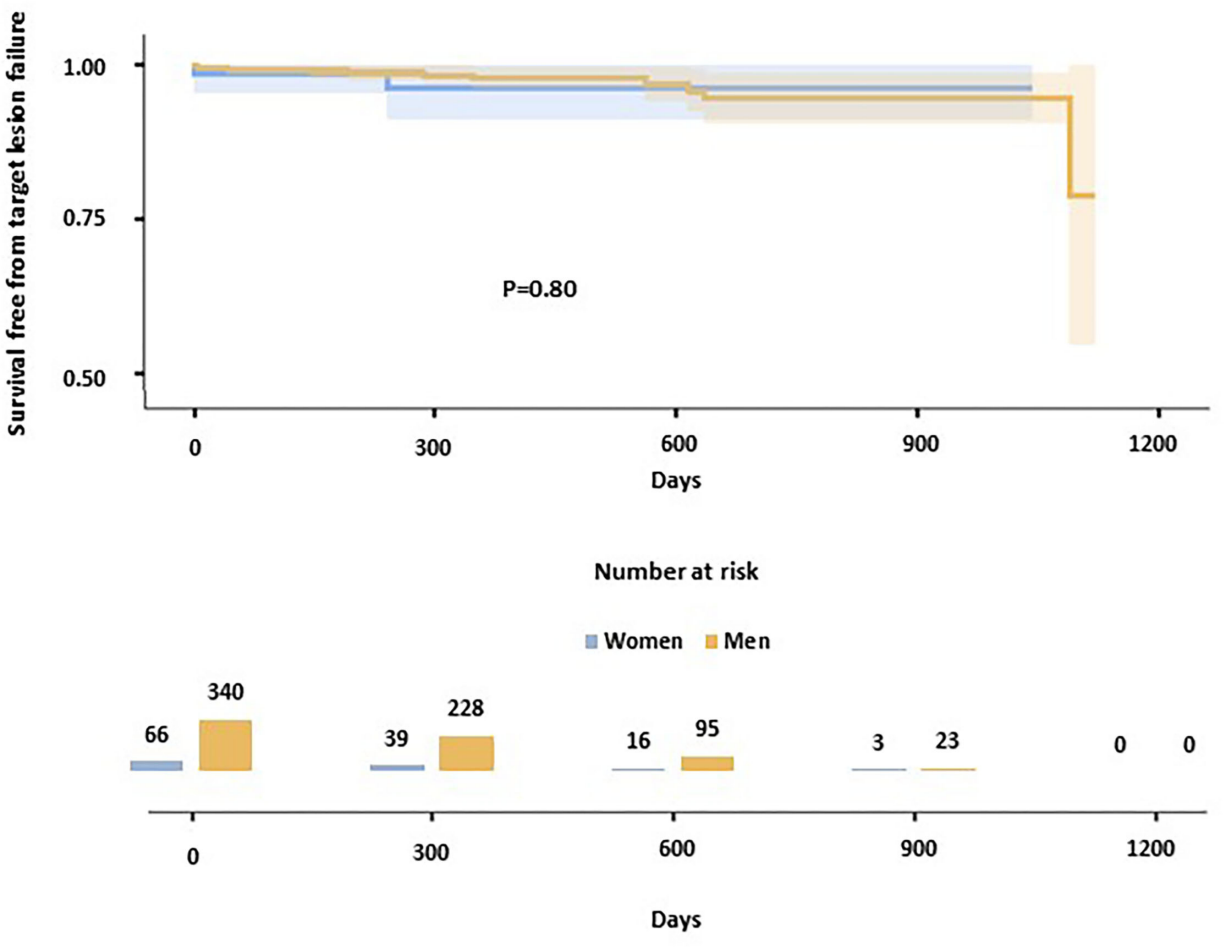
Kaplan-Meier curves show the survival-free rate from target lesion failure in the study population. Women are represented in blue, men in yellow.

After a median follow-up of 402 days (IQR 256-618), TLF occurred in 12 (2.9%) patients (i.e., 2.37 events per 100 patient-years; Table 3). No significant differences were observed among gender in terms of TLF at follow-up [10 (2.9%) in men, 2 (3.0%) in women; i.e., 2.34 events per 100 patient-years in men, 2.53 in women; *P* = 0.80 at log-rank; Figure 1]. ST, one early and one late occurred in two patients, both men. TLF occurred in 8 (4.6%) patients in the small vessel PCI group (*P* = 0.075 compared to the general population), with no significant differences between men and women (4.9% in men, 3.2% in women; *P* = 0.68). In patients with ACS, 4 TLF occurred out of 86 patients (4.7%; *P* = 0.27 compared to the general population), with no significant differences between women and men (4.3% in men, 6.3% in women; *P* = 0.74).

**Table 3:** Long-term outcomes.

	**Overall**	**Men**	**Women**	***P*-value**
	**(*N* = 415)**	**(*N* = 349)**	**(*N* = 66)**	
Target lesion failure, *n* (%)	12 (2.9)	10 (2.9)	2 (3.0)	0.94
Cardiovascular death, *n* (%)	3 (0.8)	2 (0.6)	1 (1.5)	0.41
Target vessel myocardial infarction, *n* (%)	3 (0.8)	3 (0.9)	0	1.0
Target vessel revascularization, *n* (%)	8 (1.9)	7 (2.0)	1 (1.5)	0.79
Stent thrombosis, *n* (%)	2 (0.5)	2 (0.6)	0	1.0

*Values are expressed as number (%)*.

## Discussion

The main finding of the present study is that third-generation, ultrathin BP-SES stents are associated with favorable outcomes in terms of both safety and effectiveness in the general population, irrespective of gender.

The demonstration that men and women experience similar outcomes when treated with BP-SES is a relevant clinical finding, as issues related to gender misrepresentation in clinical studies in the cardiovascular field have been known for years. Scott and colleagues have shown that RCTs for the Food and Drug Administration (FDA) approval of cardiovascular drugs included a significantly lower ratio of enrolled women compared to the diseased population, in particular for ACS and coronary artery syndromes. They calculated the participation to prevalence ratio, i.e., the ratio between the percentage of women in trial participants and the percentage of women in the disease population, and found it to be as low as 0.6 (1). Gender misrepresentation is not only an ethical issue but also a clinical issue. Several studies investigating earlier generation DES reported a higher rate of adverse stent-related events in women compared to men (4, 15). More recently, different studies have shown that women might experience worse cardiovascular outcomes not directly caused by stent-related events. A pooled analysis of two studies including patients treated with platinum-chromium second-generation everolimus-eluting stents (EES) has shown that, even after treatment with the same coronary stent, long-term outcomes might differ between women and men (5). A large analysis including patients treated with EES for left main PCI showed that women experienced a higher rate of major adverse cardiovascular events at follow-up, mainly because of higher mortality, with no substantial differences in terms of TLR (16). Also, the recent Excel trial assessing the use of second-generation EES for the left main disease has shown a somehow worse prognosis in female patients, even if this finding was not confirmed after controlling for potential confounding. Even if inconsistent (17), these findings underline the importance of focusing on potential gender-related differential outcomes in experimental settings in order to define gender-tailored treatment strategies.

The third-generation ultrathin BP-SES included in our analysis has shown excellent results in RCTs in terms of effectiveness and safety. It outperformed durable polymer EES consistently across different populations (10) and different clinical presentations, including STEMI (18, 19). The low rate of TLF reported in our study, reflecting real-world evidence of TLR rates in unselected patients, is similar to the one reported by other studies, such as the Canadian sub-analysis from the Bioflow-III registry (20), but is higher compared to the one by other registries, such as the Bioflow-III registry main analysis or the Italian subgroup analysis (21, 22). These findings may be explained by the relatively low rate of patients with ACS included in our analysis, which reflects the large elective activity performed at our institution, as about 20% of the patients included underwent PCI for ACS, compared to 52.7% in the main analysis of Bioflow-III registry. Of interest, in our study, more than 40% of the patients underwent small coronary vessel PCI: even if small stent diameter has been known as a predictor of restenosis (23) and ST (24) for years, results from previous studies with the ultra-thin BP-SES have shown no significant differences in terms of TLF at follow-up (22). Our study showed, despite not statistically significant, a trend toward a higher rate of TLF in patients with small vessel PCI compared to the general population, even if these results did not reach statistical significance, without differences between men and women. Similar results were observed for patients presenting with ACS. Given their potential clinical rebounds, these results need further monitoring and investigation in futures real-world registries.

### Study Limitations

The main limitation of the present study is the retrospective design, which does not allow to control for unaccounted confounding. Moreover, the choice to implant the investigated BP-SES was left at the operator's discretion and not guided by the study protocol. The low number of adverse events recorded did not allow to perform a multivariate adjustment.

## Conclusion

Third-generation, ultrathin BP-SES does not appear to be associated with an increased risk of adverse outcomes in a real-world clinical setting, irrespective of the gender of the treated patients. Continuous post-marketing monitoring and larger-scale studies are needed to corroborate these positive results.

## Data Availability Statement

The datasets presented in this study can be found in online repositories. The names of the repository/repositories and accession number(s) can be found at: https://zenodo.org/record/5236411#.YXbcXBrMKUk.

## Ethics Statement

The studies involving human participants were reviewed and approved by Centro Cardiologico Monzino, IRCCS Ethic Committee. The patients/participants provided their written informed consent to participate in this study.

## Author Contributions

SGi and DT: conceptualization and writing. PM, FF, AB, and PR: revision. GT, GS, and SGa: data collection. SGi: statistical analysis. DT: data analysis and revision. All authors contributed to the article and approved the submitted version.

## Conflict of Interest

The authors declare that the research was conducted in the absence of any commercial or financial relationships that could be construed as a potential conflict of interest.

## Publisher's Note

All claims expressed in this article are solely those of the authors and do not necessarily represent those of their affiliated organizations, or those of the publisher, the editors and the reviewers. Any product that may be evaluated in this article, or claim that may be made by its manufacturer, is not guaranteed or endorsed by the publisher.
